# Russeting in apple and pear: a plastic periderm replaces a stiff cuticle

**DOI:** 10.1093/aobpla/pls048

**Published:** 2012-12-17

**Authors:** Bishnu P. Khanal, Eckhard Grimm, Moritz Knoche

**Affiliations:** Institute for Biological Production Systems, Fruit Science Section, Leibniz University Hannover, Herrenhäuser Straße 2, 30419 Hannover, Germany

**Keywords:** cuticular membrane, fracture, fruit skin, mechanical properties, rheology, russet, strain

## Abstract

**Background and aims:**

Russeting in apples (*Malus* × *domestica* Borkh.) and pears (*Pyrus communis* L.) is a disorder of the fruit skin that results from microscopic cracks in the cuticle and the subsequent formation of a periderm. To better understand russeting, rheological properties of cuticular membranes (CM) and periderm membranes (PM) were studied from the russet-sensitive apple ‘Karmijn de Sonnaville’ and from ‘Conference’ pear.

**Methodology:**

The CM and PM were isolated enzymatically, investigated by microscopy and subjected to tensile tests, creep/relaxation tests and to stepwise creep tests using a material testing machine.

**Principal results:**

The isolated CM formed a continuous polymer, whereas the PM represented a cellular structure of stacked cork cells. Tensile tests revealed higher plasticity of the hydrated PM compared with the CM, as indicated by a higher strain at the maximum force (*ɛ*_max_) and a lower modulus of elasticity (*E*). In apple, the maximum force (*F*_max_) was higher in the CM than in the PM but in pear the higher *F*_max_ value was found for the PM. In specimens obtained from the CM : PM transition zone, the weak point in apple was found to be at the CM : PM borderline but in pear it was within the CM. In both apple and pear, creep/relaxation tests revealed elastic strain, creep strain, viscoelastic strain and viscous strain components in both the PM and CM. For any particular force, strains were always greater in the PM than in the CM and were also greater in pear than in apple. The *ɛ*_max_ and *F*_max_ values of the CM and PM were lower than those of non-russeted and russeted whole-fruit skin segments, which included adhering tissue.

**Conclusions:**

In russeting, stiff CM are replaced by more plastic PM. Further, the cell layers underlying the CM and PM represent the load-bearing structure in the fruit skin in apple and pear.

## Abstract (precis)

In russeting of apple and pear fruit, a stiff cuticle is replaced by a more plastic periderm. Furthermore, the cell layers underlying the cuticle and the periderm represent the load-bearing structure in the fruit skin in both apple and pear.

## Introduction

Russeting is a commercially important surface defect in many fruit crops including apples and pears, with russeted fruit often having reduced market value (Faust and Shear 1972*a*; Wertheim 1982). In functional terms, russeting restores control of water loss through the skin by the formation of a waterproofing periderm (phellem, phellogen and phelloderm) just beneath the microcracked primary fruit skin (cuticle, epidermis and hypodermis).

Studies on the aetiology of russeting identify the formation of microscopic cracks in the primary fruit skin as the first visible sign of russeting (for reviews see Faust and Shear 1972*a*, *b*). These microcracks apparently trigger formation of a periderm (Faust and Shear 1972*a*). Microscopy reveals that the periderm is differentiated in the subepidermal cell layers underlying the cracks and the epidermis (Verner 1938; Meyer 1944). Later, the primary surface comprising cuticle and epidermis dries and is shed, and the phellem of the secondary surface becomes visible as the familiar ‘russeting’. It is the phellem at the new surface that is responsible for the dull, rough, brown, corky appearance of a russeted fruit.

Growth stresses are considered to be the driving force for the formation of microcracks (Skene 1982). The increase in fruit volume during development subjects the skin to biaxial tangential strain and stress. Failure occurs when the extensibility limits of the skin are exceeded. Strain and stress are greatest in the outermost layers of a fruit. They peak when relative area growth rates are maximal. For a fruit such as the apple, which has a sigmoid growth pattern, the area growth peak occurs in the early phase of development in the period up to ∼3 weeks after full bloom. Observations show that apples are most sensitive to russeting at this time (Wertheim 1982; Knoche *et al.* 2011). Additional factors contributing to microcracking are: (i) inhomogeneous surface expansion resulting from irregular cell division in the epidermal or hypodermal cell layers (Eccher 1975), (ii) increases in the turgor of dividing and expanding epidermal cells (Faust and Shear 1972*a*), (iii) extended periods of surface wetness or high humidity (Tukey 1969; Knoche *et al.* 2011) and (iv) a mismatch between surface expansion and cuticle deposition in many soft and fleshy fruit crops, such as sweet cherries (Knoche *et al.* 2004), various *Ribes* berries (Khanal *et al.* 2011) and grapes (Becker and Knoche 2012).

While the phenomenological sequence of events in russeting is largely established (Verner 1938; Faust and Shear 1972*a*), little is known about the mechanical properties of the load-bearing structures in primary and secondary fruit skins (Petracek and Bukovac 1995; Bargel and Neinhuis 2004; Dominguez *et al.* 2011). Of particular interest are the mechanical characteristics of the outermost layers of the fruit skin, because here strain and stress are maximal. These layers include the cuticle of the primary skin of non-russeted fruit and the periderm or the secondary skin of russeted fruit. For recent reviews on the chemistry of major constituents of cuticle and periderm, the reader is referred to Dominguez *et al.* (2011), Heredia (2003) and Franke and Schreiber (2007).

The objectives of the present study were to characterize the rheological properties of fruit cuticles and periderms, and to measure their failure thresholds. Also, we aimed to identify the site of failure of composite fruit-skin specimens comprising both cuticle and periderm to determine whether russeting increases due to (i) the ‘spreading’ of russeting as a result of failure within a russeted area or at the boundary between a russeted and a non-russeted area or (ii) the formation of new sites of russeting because of failure in a non-russeted area. For our studies, we employed uniaxial mechanical tests of the isolated cuticular membrane (CM) and periderm membrane (PM), and ‘composite’ fruit skins comprising CM and PM (CM/PM) from apple and pear as a model.

## Methods

### Plant material

Fruit of the apple (*Malus* *×* *domestica* Borkh.) ‘Karmijn de Sonnaville’ (hereafter referred to as ‘Karmijn’) and the pear (*Pyrus communis* L.) ‘Conference’ were obtained at commercial maturity from the experimental orchards (52°14′N, 9°49′E) of Leibniz University, Hannover, Germany. Fruit were grown according to the European Union regulations for integrated fruit production and harvested at commercial maturity. Unless otherwise specified, fruit that were used to supply CM and PM samples were stored in either conventional cold storage or controlled atmosphere storage for up to 8 months, and those serving as a source of epidermal segments (ES) and peridermal segments (PS) were taken from freshly harvested fruit.

### Preparation of the ES and PS and isolation of the CM and PM

Segments of the fruit skin were excised from russeted, non-russeted or russeted/non-russeted transition regions (∼50 % each) of apples and pears using a cork borer (24 mm inner diameter) or a custom-made punch that produces a biconcave (dumb-bell-shaped) specimen with a narrow waist (width 4.25 mm). To minimize natural curvature, samples were taken from the equatorial region of the fruit (minimum radius of curvature). Samples were used either fresh as ES when excised from non-russeted skins or as PS when excised from russeted skins. For the preparation of CM and PM, the samples were incubated in 50 mM citric acid buffer solution (pH 4.0) containing pectinase (90 mL L^−1^, Panzym Super E flüssig; Novozymes A/S, Krogshoejvej, Bagsvaerd, Denmark), cellulase (5 mL L^−1^, Cellubrix L; Novozymes A/S) and NaN_3_ at 30 mM (Orgell 1955; Yamada *et al.* 1964; Groh *et al*. 2002). The isolation medium was refreshed periodically until the CM, PM or CM/PM separated from the underlying tissue. Specimens were then rinsed with deionized water and dried at ambient temperature and humidity (22 °C and 50 % relative humidity (RH)). The average mass per unit surface area was quantified using five replicates comprising six discs each.

### Scanning electron and fluorescence light microscopy

Freeze-fractured CM and PM samples were prepared for scanning electron microscopy. Specimens were mounted on aluminium stubs using conducting (carbon) tape and viewed under a scanning electron microscope (Quanta 200; FEI Europe Main Office, Eindhoven, The Netherlands) at ×1200, an acceleration potential of 10 kV and a pressure of 60 Pa.

Samples of the isolated CM and PM were also inspected using a fluorescence microscope (BX60 with filter U-MWU, 330−385 nm excitation, ≥420 nm emission; Olympus Europa Holding GmbH, Hamburg, Germany) and a dissecting microscope (MZ10F with filter GFP-plus, 440−480 nm excitation, ≥510 nm emission; Leica Microsysteme GmbH, Wetzlar, Germany). Micrographs were taken using a digital camera (DP71, Olympus; Software Cell^P, Olympus).

### Strain of the CM and PM

The release of biaxial strain of the CM and PM following excision and isolation was quantified using epidermal and peridermal discs excised from freshly harvested apple and pear fruit. Before excision, a square pattern (7 × 7 mm) of four holes (0.55 mm diameter) was punched in the non-russeted and russeted surfaces in the equatorial region of the fruit using a custom-made punch. The area (*A*; mm^2^) enclosed by the hole pattern in the ES (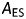) and PS (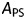) was quantified by light microscopy and image analysis (Cell^P). Subsequently, the epidermal and peridermal discs were excised and the CM and PM were isolated enzymatically as described above. The isolated discs were then mounted on microscope slides and re-photographed. The areas enclosed by the hole pattern on the CM (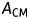) and PM (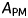) were re-quantified. The release of biaxial strain (*ɛ*, %) was calculated (Knoche *et al.* 2011) as
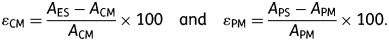



### Mechanical tests

Strips (5 mm wide) were prepared from enzymatically isolated CM, PM and CM/PM discs using parallel razor blades. To facilitate handling during preparation and mounting, the strips were fixed in a frame made of paper and masking tape (Tesa Krepp^®^; Tesa Werk Hamburg GmbH, Hamburg, Germany). Unless specified otherwise, tensile tests were performed with both dry and hydrated specimens. Dry specimens were held at 50 % RH and 22 °C (‘dry’), and hydrated specimens were preconditioned overnight by incubating in deionized water at 22 °C (‘hydrated’). Thereafter, frames were mounted in a universal material testing machine (Z 0.5; Zwick/Roell, Ulm, Germany; clamping distance 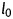 = 10 mm) equipped with a 10 N force transducer (KAP-Z; Zwick/Roell). Frames were cut open and the following experiments were conducted.

Uniaxial tensile tests were performed at a strain rate of 1 mm min^−1^ until failure of the specimen with tensile force (*F*; newton) and crosshead displacement (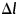; mm) being recorded. Uniaxial strains (*ɛ*; %) were calculated by dividing 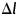 by the initial length of the specimen (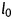; mm):
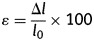



The maximum force (*F*_max_; newton) and the strain at the maximum force (*ɛ*_max_; %) were recorded. The modulus of elasticity (*E*; newton) was calculated as the maximum slope of a linear regression line fitted through a plot of force (newton) vs. strain (*ɛ*/100). Following tensile tests, surface views of fractured apple CM were inspected by light microscopy (BX60) at ×10. The lengths of the fracture were quantified in the two fracture-mode categories, ‘fracture along cell walls’ and ‘fracture across cell walls’, using image analysis (Cell^P). In apple, only ∼10 % of the fracture length could not be assigned unambiguously to one or the other of these fracture modes.

**Fig. 1:**
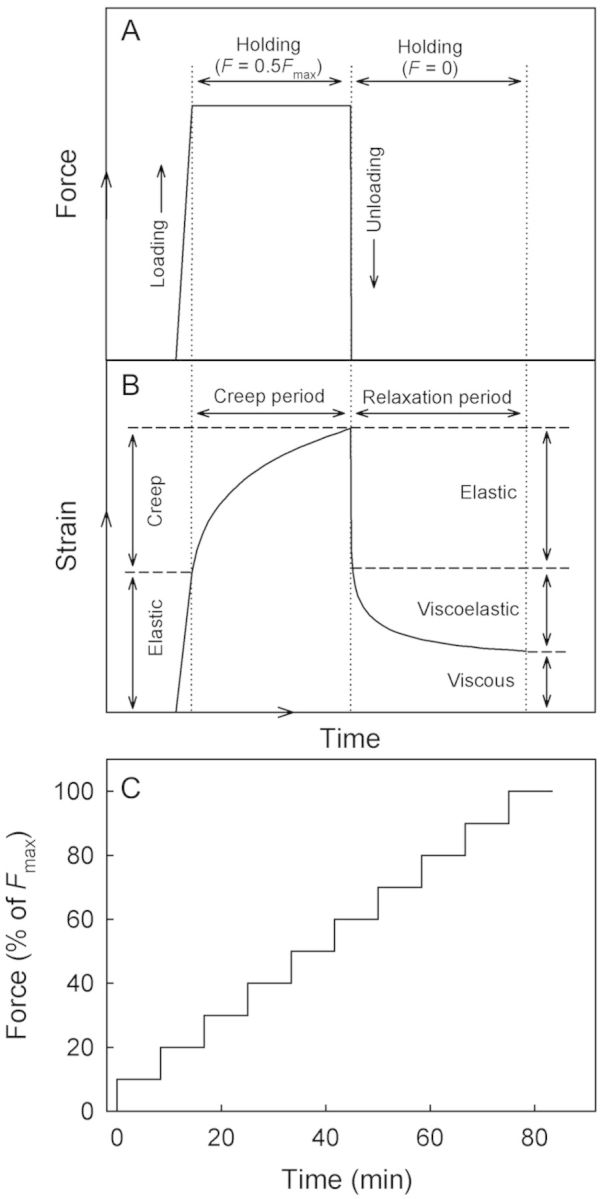
**(A and B) Schematic model of the creep/relaxation test used to quantify elastic strain and creep strain during the loading cycle, and elastic strain, viscoelastic strain and viscous strain during the unloading cycle of excised and enzymatically isolated CM and PM of apples and pears.** During the loading cycle, specimens were subjected to tensile forces that equalled 50 % of the respective maximum forces (*F*_max_). (C) Sketch of stepwise creep tests where CM and PM strips of apple and pear were loaded stepwise in increments of 10 % of their respective *F*_max_ values.

A creep/relaxation test composed of a loading and an unloading cycle to monitor creep and creep relaxation, respectively, was performed on the hydrated CM and PM from apples and pears (Fig. 1A and B). During loading, a force equivalent to 0.5*F*_max_ of the respective specimen (*F*_max_ in apple: 1.00 ± 0.04 N for the CM and 0.59 ± 0.04 N for the PM; *F*_max_ in pear: 0.27 ± 0.01 N for the CM and 0.53 ± 0.03 N for the PM) was applied at a rate of 0.5 mm min^−1^ followed by a 500-s hold period (Fig. 1A). The instantaneous elastic strain during loading was calculated as the relative increase in specimen length during the period of force application (Fig. 1A and B). In the subsequent hold period (force constant), the specimen extended due to creep (Fig. 1A and B). The strain occurring during the hold period is referred to as the creep strain. This creep strain was calculated as the relative increase in specimen length during the hold period of constant force (Meyers and Chawla 2009; Fig. 1B). In the subsequent unloading cycle, the specimen contracted almost instantaneously upon force removal followed by a time-dependent viscoelastic contraction. The instantaneous elastic contraction corresponds to the release of elastic strain, and the subsequent time-dependent viscoelastic contraction to the release of viscoelastic strain (Fig. 1A and B). Elastic and viscoelastic strains during unloading were calculated as the respective percentage decreases in length of the specimen (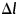) divided by the initial clamping distance (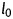). The extension of the specimen that remained after 1000 s from initiation of the experiment represents the plastic or viscous irreversible strain (Fig. 1B). During testing, the hydration state of the specimens was maintained by misting (Pariboy; Pari GmbH, Starnberg, Germany) and running deionized water over the specimen (Perfusor^®^ Compact S, B BRAUN, Melsungen, Germany). The number of replications was 9 or 10.

The effect of stepwise force increases on creep strain was monitored in a stepwise creep test using fully hydrated apple and pear CM and PM (Fig. 1C). Preliminary experiments were conducted to quantify the *F*_max_ of the dry and hydrated apple CM (dry: 1.22 ± 0.05 N, hydrated: 0.83 ± 0.05 N) and pear CM (dry: 0.42 ± 0.03 N, hydrated: 0.27 ± 0.01 N) and dry and hydrated apple PM (dry: 1.27 ± 0.06 N, hydrated: 0.66 ± 0.02 N) and pear PM (dry: 0.81 ± 0.07 N, hydrated: 0.53 ± 0.03 N). Creep tests were performed by increasing the force stepwise in increments of *F*_max_/10 each step followed by a 500-s holding period (Fig. 1C). Increase was continued until the specimen failed. The crosshead speed during force applications was constant at 0.5 mm min^−1^. Specimens remained fully hydrated throughout the test. Force, crosshead travel and time were recorded. The elastic strain and the creep strain were quantified as described above.

To relate the rheological properties and fracture thresholds determined in the CM and PM to those of intact russeted and non-russeted fruit skins, uniaxial tensile tests were also performed on the ES and PS. Biconcave, dumb-bell-shaped specimens (waist width 4.25 mm) were excised from non-russeted or russeted regions of the cheek of fresh apple and pear fruit using the custom-made punch. The long axis of all specimens was oriented longitudinally (i.e. parallel to the calyx/pedicel axis). Preliminary experiments established that there was little difference in the mechanical properties of the ES excised in longitudinal and latitudinal orientations, implying that specimens were approximately isotropic (B. P. Khanal, unpubl. observ.). The ES and PS were carved by hand to varying thicknesses using a razor blade and a simple jig. The thickness of ES and PS samples was quantified by light microscopy. Uniaxial tensile tests were performed as described above (Z 0.5; Zwick/Roell; 50 N force transducer, Type: KAP-TC; Zwick/Roell). Clamping distance was 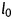 = 16 mm and the crosshead speed 3 mm min^−1^. There was no preconditioning of the specimen and all tests were completed within 3 min of excision. The *F*_max_ and *ɛ*_max_ values were measured. For comparison, enzymatically isolated CM and PM strips from the same batch of fruit were also investigated.

### Data analysis and terminology

Occasionally, CM, PM or CM/PM specimens failed in, or adjacent to, the clamps. It is thought likely that such specimens may have been damaged during preparation and/or clamping and these data were therefore excluded from the analyses. The specimens not excluded in this manner represented 84, 81 and 91 % of the total population of CM, CM/PM and PM measurements, respectively. None of the ES or PS specimens failed in, or adjacent to, the clamps. Analysis of variance (ANOVA) and regression analyses were performed using SAS (version 9.1.3; SAS Institute, Cary, NC, USA). Values expressed on a percentage basis were arcsine transformed before ANOVA. Unless individual observations are shown, values in the figures are presented as the means ± SE of means. Where not visible, the error bars are smaller than the plotting symbols.

Throughout the article, the fruit skin segments excised from non-russeted and russeted regions of the fruit surface are referred to as epidermal segments (ES) and peridermal segments (PS), respectively. The specimens obtained after enzymatic isolation of the ES and PS are referred to as the cuticular membranes (CM) or periderm membranes (PM).

## Results

### Microscopic structure of the CM and PM

**Fig. 2:**
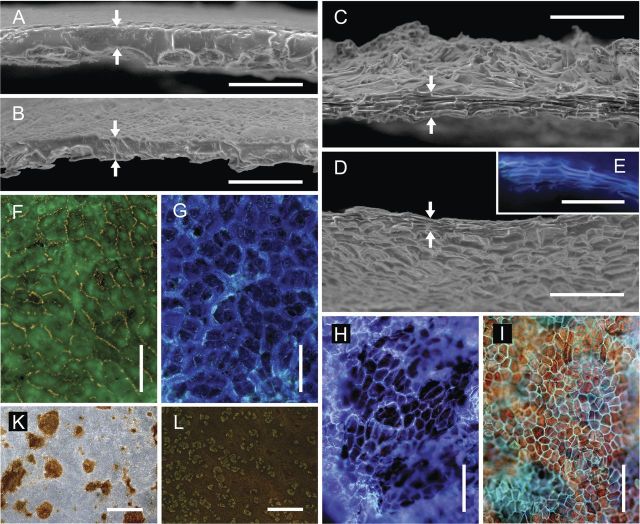
**Scanning electron (A–D) and fluorescence light micrographs (E–L) of the enzymatically isolated CM (A, B, F, G and K) and PM (C, D, E, H, I and L) excised and isolated from non-russeted and russeted regions of apple (A, C, F, G and L) and pear fruit surfaces (B, D, E, H, I and K).** Cross-sections obtained by freeze fracture of the CM and PM (A–E), the CM and PM viewed from above (F, H, K and L) and below (G and I) under incident UV light (filter U-MWU; E, F, G, H and I; filter GFP-plus, L), and transmitted (F–I) and incident (K) white light. Upper and lower edges of the freeze-fractured surface are indicated by arrows. Scale bars: (A–E) 0.05 mm; (F–I) 0.1 mm; (K and L) 2 mm.

Scanning electron microscopy of cross-sections of the CM revealed a continuous cuticular lamella above the former periclinal epidermal cell walls (Fig. 2A and B) and cuticular pegs of variable size above the former anticlinal epidermal cell walls (Fig. 2A, B, F and G). The larger and thicker pegs separated imprints of groups of epidermal cells (Fig. 2F and G), while the smaller pegs provided a substructure within these groups, separating imprints of the individual epidermal cells (Fig. 2G). The CM of apple and pear often contained spots of periderm, as indicated by the brown colour and cellular structure when viewed from the morphological outer side (Fig. 2K). The percentage of surface area with periderm in the CM was somewhat higher in pear than in apple (E. Grimm, unpubl. observ.).

In contrast to the CM, the structure of the PM in apple and in pear was cellular (Fig. 2C, D, E, H, I and L). Cross-sections revealed stacked phellem cells, all elongated tangentially (Fig. 2C–E). When inspected from above or below, phellem cells were polygonal, isodiametric, with no preferential longitudinal or latitudinal orientation (Fig. 2C, D, H and I). Cell walls of the phellem cells were encrusted with suberin, as indicated by autofluorescence (Fig. 2E, H, I and L). In cross-sections, patches of the CM and epidermis overlaying the periderm were observed, which indicates that the PM was formed within the subepidermal tissue (Fig. 2L).

In both fruit types, the mass per unit area of the CM was significantly higher than that of the PM (apple, +26 %; pear, +34 %) (Table 1). Isolates obtained from transition zones between the CM and PM had mass per area values intermediate between those of the CM and PM (results not shown).

**Table 1:** **Physical properties of CM and PM discs excised and isolated from mature ‘Karmijn de Sonnaville’ apples and ‘Conference’ pears.** Biaxial strain release was calculated as a fractional (%) decrease in the area of the CM and PM upon excision and enzymatic isolation.

Specimen	Mass (g m^−2^)		Strain (%)	
	Apple	Pear	Apple	Pear
CM	23.7 ± 0.4a*	17.3 ± 0.4a	5.7 ± 0.4b	7.6 ± 0.5b
PM	17.4 ± 0.4b	11.7 ± 0.2b	11.0 ± 0.6a	21.8 ± 1.1a

*Data are the means and standard errors of five replicates (comprising six CM or PM discs each) for mass and of 30 replicates for strain.

Mean separation within species by Tukey's Studentized range test, *P* < 0.05.

After excision and enzymatic isolation, the areas of both CM and PM discs were smaller than before processing, indicating that strain release had occurred. The release of strain from PM discs exceeded that from CM discs (1.9-fold in apple; 2.9-fold in pear) (Table 1).

### Mechanical tests

**Fig. 3:**
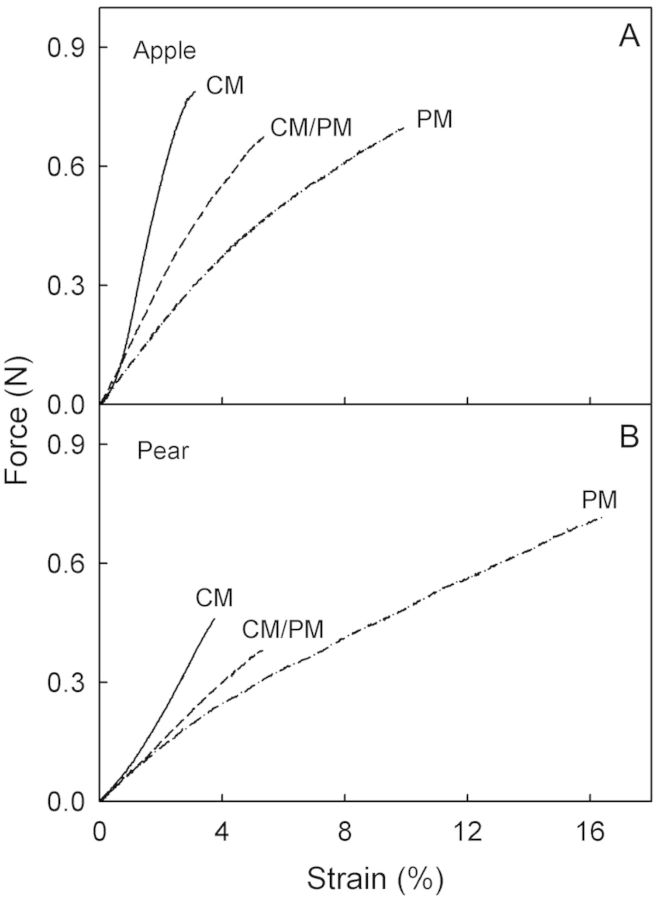
**Representative force/strain diagrams of hydrated CM, PM and membranes with CM/PM transition zones subjected to a uniaxial tensile test.** Specimens were enzymatically isolated from non-russeted (CM), russeted (PM) and transition regions (CM/PM) of apple (A) and pear skins (B). For the maximum force, strain at the maximum force and modulus of elasticity, see Table 2.

Tensile tests of strips of hydrated CM, PM and of CM/PM transition zones revealed qualitatively similar force–strain relationships (Fig. 3; Table 2). The maximum force (*F*_max_) was lower in the PM than in the CM in apple, but higher in the PM than in the CM in pear. The *ɛ*_max_ values were markedly higher in the PM than in the CM or the CM/PM (Table 2) and, hence, the *E* values were lower. When wet and dry specimens were compared, the hydrated CM, PM or CM/PM generally had lower *F*_max_ and *E* values than their dry counterparts (Table 2). Furthermore, the hydrated PM had higher *ɛ*_max_ values than the dry PM. However, there was little effect of hydration on the CM. Quantitatively similar results had been obtained in an earlier season (B. P. Khanal, unpubl. observ.) reinforcing this observation.

**Table 2:** **Maximum force (*F*_max_), strain at maximum force (*ɛ*_max_) and modulus of elasticity (*E*) of CM and PM isolated enzymatically from the fruit skins of mature ‘Karmijn de Sonnaville’ apples and ‘Conference’ pears.** CM/PM denotes specimens that were excised and isolated from a transition zone between the CM and PM. Approximately half of the specimen was composed of CM and the other half of PM. Specimens were subjected to uniaxial tensile tests in either a dry or a hydrated state.

Species	State	*F*_max_ (N)			*ɛ*_max_ (%)			*E* (N)		
		CM	CM/PM	PM	CM	CM/PM	PM	CM	CM/PM	PM
Apple	Dry	1.22 ± 0.05ab*	1.09 ± 0.04b	1.27 ± 0.06a	3.9 ± 0.3a	3.5 ± 0.1a	4.1 ± 0.2a	49.8 ± 0.5a	36.9 ± 0.8b	36.9 ± 1.0b
	Hydrated	0.83 ± 0.05a	0.66 ± 0.03b	0.66 ± 0.02b	2.9 ± 0.2c	5.5 ± 0.4b	9.6 ± 0.4a	39.1 ± 1.3a	17.0 ± 0.6b	10.6 ± 0.5c
Pear	Dry	0.41 ± 0.02b	0.33 ± 0.02b	1.09 ± 0.05a	3.5 ± 0.1b	3.2 ± 0.2b	7.4 ± 0.2a	13.9 ± 0.6b	12.1 ± 0.7b	17.8 ± 1.0a
	Hydrated	0.40 ± 0.02b	0.31 ± 0.03c	0.65 ± 0.02a	3.4 ± 0.2c	5.5 ± 0.4b	16.5 ± 0.5a	15.2 ± 0.9a	6.4 ± 0.5b	5.5 ± 0.4b

*Values represent the means and standard errors of a minimum of 14 replicates (apple) and 16 replicates (pear). Interaction between the state of hydration and type of specimen significant in two-factorial ANOVA for apple and pear (*P* < 0.0001 for *F*_max_, *ɛ*_max_ and *E* in apple and pear except for *F*_max_ in apple where *P* < 0.0663). Therefore, one-factorial ANOVA was run and mean comparisons were made within species and state of hydration using Tukey's Studentized range test, *P* < 0.05.

The site of failure of specimens from the CM/PM transition zone differed between apple and pear. In apple, failure was more frequent at the CM/PM transition, compared with failure at the PM or the CM (Table 3). In pear, however, segments failed more often at the CM portion, followed by the CM/PM transition zone or the PM portion (Table 3). The site of failure was not affected by the hydration state.

**Table 3:** **Frequency of fracture in different regions of specimens excised from apple (‘Karmijn de Sonnaville’) and pear (‘Conference’) fruit surfaces.** The specimens selected had a transition zone between the russeted portion with a PM and a non-russeted portion with a CM. Following enzymatic isolation, the specimens were subjected to uniaxial tensile tests, the position of failure (CM vs. CM/PM vs. PM) was noted, and the failure frequency (%) was calculated.

Species	State	*n*	Failure frequency (%)		
			CM	CM/PM	PM
Apple	Dry	27	14.8	51.9	33.3
	Hydrated	20	15.0	50.0	35.0
Pear	Dry	20	65.0	35.0	0
	Hydrated	17	82.4	11.8	5.9

Microscopic inspection of the fracture surfaces obtained in tensile tests of the apple CM revealed that failure occurred mostly along the cuticular pegs and thus along the anticlinal cell walls (56 % of total fracture length; data not shown). Failure occurred less across the pegs and, hence, across the cell walls (34 % of the total fracture length). On average, the optical resolution was insufficient to assess the site of failure over 10 % of the total fracture length.

**Fig. 4:**
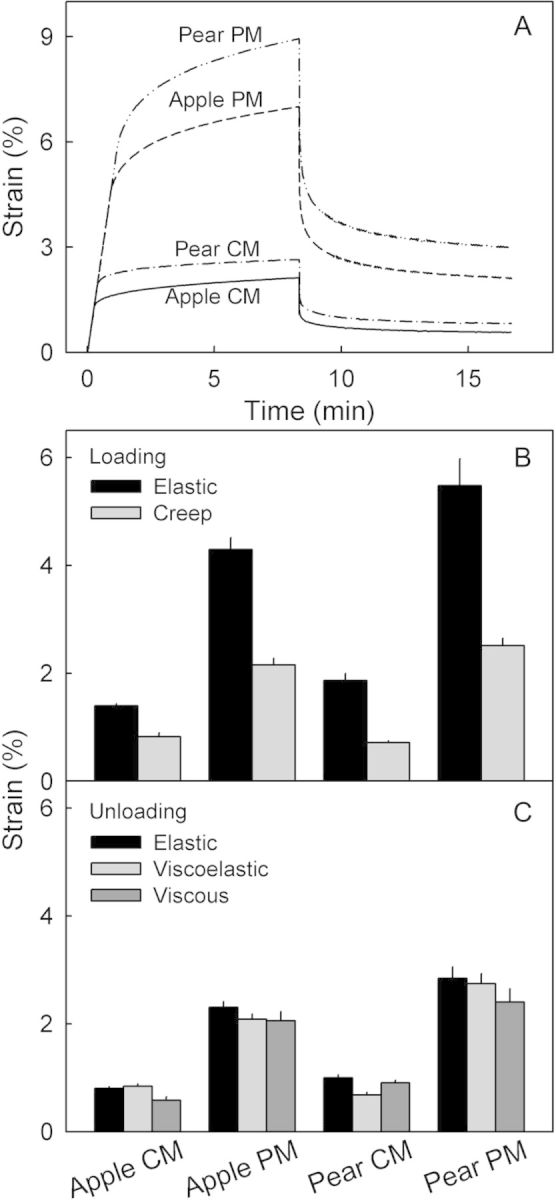
**Creep/relaxation test of CM and PM enzymatically isolated from non-russeted and russeted surfaces of mature ‘Karmijn de Sonnaville’ apple and ‘Conference’ pear fruit.** (A) Representative time courses of strain during the loading and the unloading cycle. (B; *n* = 9–10) Elastic and creep strain during the loading cycle. (C; *n* = 9–10) Elastic strain, viscoelastic strain and viscous strain during the unloading cycle. For details see the text.

Creep/relaxation tests of the CM and PM for both apple and pear consistently yielded qualitatively similar but quantitatively different changes in strain with force and time (Fig. 4A). During loading, elastic strain and creep strain values in the PM exceeded those in the CM. Both sorts of strain were of similar magnitude in apple and pear (Fig. 4B). During unloading, the release of elastic strain and viscoelastic strain from the PM and the remaining viscous strain of the PM were all larger than those from the CM (Fig. 4C). There was little difference between apple and pear in this regard. A comparison of loading and unloading cycles revealed that the elastic strain of the CM and PM during loading exceeded the elastic strain released during unloading.

**Fig. 5:**
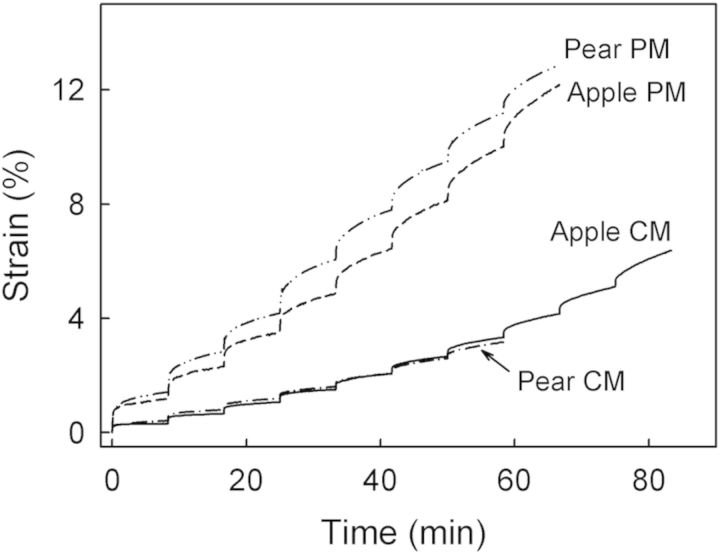
**Representative time courses of a stepwise creep test performed on CM and PM enzymatically isolated from non-russeted and russeted surfaces of mature ‘Karmijn de Sonnaville’ apple and ‘Conference’ pear fruit.** All specimens were fully hydrated when tested.

**Fig. 6:**
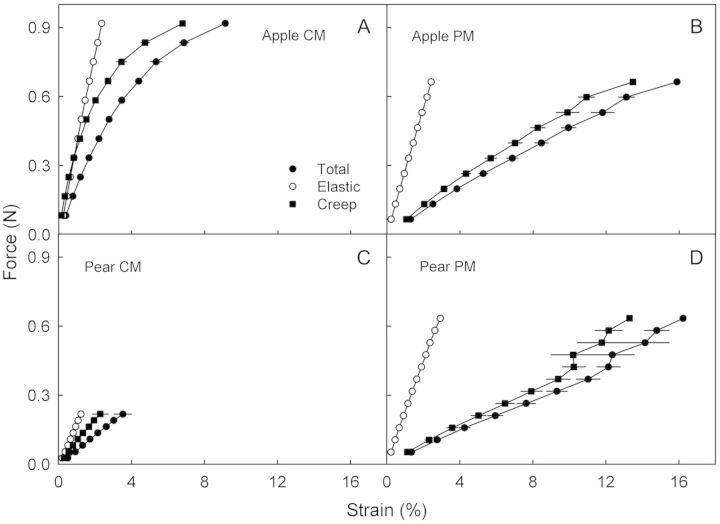
**Force/strain diagrams of CM and PM enzymatically isolated from non-russeted and russeted surfaces of mature ‘Karmijn de Sonnaville’ apple and ‘Conference’ pear fruit.** The force was increased stepwise in increments of 10 % of the maximum force (*F*_max_). Each increment was followed by a 500-s hold period. Total strain was partitioned into an elastic strain and a creep strain. For details see the text. *n* = 14–16 (apple) and *n* = 10 (pear).

In the stepwise creep test, the strain in the CM increased with each force step for both apple and pear. This response was even more marked in the PM (Fig. 5). This increase is accounted for primarily by larger creep strains, which in each case exceeded the elastic strains several fold (Fig. 6). The *F*_max_ and *ɛ*_max_ values were lower in the CM of pear than in apple (Fig. 6A and C). There was little difference in *F*_max_ and *ɛ*_max_ between the PM of each species (Fig. 6B and D). Qualitatively similar results were obtained with the dry specimens (results not shown).

**Fig. 7:**
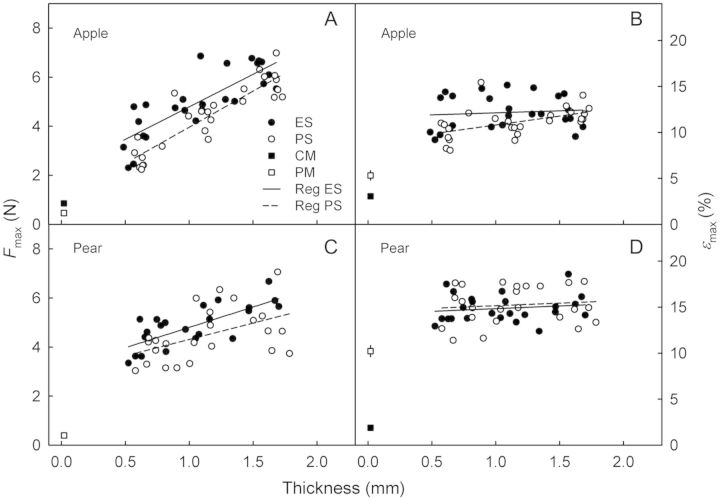
**Mechanical properties of skin segments excised from non-russeted (ES) and russeted (PS) regions of mature ‘Karmijn de Sonnaville’ apples (A and B; *n* = 26) and ‘Conference’ pears (C and D; *n* = 24).** The maximum force (*F*_max_) vs. thickness of the ES and PS (A and C), and strain at the maximum force (*ɛ*_max_) vs. thickness of the ES and PS (B and D). For comparison, the results from tensile tests of enzymatically isolated CM and PM obtained from the same batches of fruit are included. Skin thickness was determined by microscopy prior to the test.

To relate the mechanical properties of the isolated CM and PM to those of the corresponding fruit skins, the excised ES and PS were subjected to tensile tests. It was found that *F*_max_ increased linearly with thickness and, hence, with the number of parenchyma cell layers in the ES and PS in both apple and pear (Fig. 7A and C). The extrapolated *y*-axis intercepts of these relationships predict *F*_max_ values that exceed those of the isolated CM and PM in both apple and pear. The *ɛ*_max_ values of the ES and PS did not depend on thickness in apple or in pear (Fig. 7B and D). There was little difference in *ɛ*_max_ of either the ES or PS between apple and pear. The *ɛ*_max_ values of the ES and PS generally exceeded those of the corresponding CM and PM.

## Discussion

Our results demonstrate that: (i) the rheological properties of the CM and PM resemble those of viscoelastic polymers, (ii) the PM is a more plastic replacement for the stiffer CM in both apple and pear, (iii) the weak link in apple specimens obtained from russeted fruit surfaces is the borderline between the CM and PM, but in pear it is the CM, and (iv) the cell layers of the ES and PS underlying the CM and PM represent the principal load-bearing structure in both apple and pear.

### The CM and PM are viscoelastic polymers

Deformation of viscoelastic polymers typically consists of reversible elastic, time-dependent reversible viscoelastic and irreversible residual viscous or plastic components. These were all observed in the CM and PM of both apple and pear. During the loading phase in the creep/relaxation experiment, as force increased the initial deformation was mostly elastic. Creep occurred in the subsequent hold phase. This creep strain comprised viscoelastic and viscous deformation components (Niklas 1992). Attempts to partition the creep strain into viscoelastic and viscous components are ambiguous because, in our experiments, clear transitions could not be identified during the creep period (Fig. 1B). However, during the subsequent relaxation period, the time-dependent reversible deformation represents the viscoelastic strain, while the viscous strain equalled the time-independent irreversible strain (Niklas 1992; Fig. 1B).

Some differences between the force/strain behaviour of the viscoelastic CM and PM and that of the viscoelastic cell wall described by Niklas (1992) were observed. First, the release of elastic strain during unloading was only about half of the elastic strain occurring during loading (Fig. 4). Second, the sum of the viscoelastic and viscous strains during relaxation was about twice the creep strains during the hold period (Fig. 4). In the Niklas (1992) system, the sum of viscoelastic and viscous strains during unloading equalled the creep strain during loading. The reasons for these differences are unknown.

### The PM is a plastic replacement for the stiffer CM

We now focus on the hydrated CM and PM because these reflect the *in vivo* condition in which the inner side of the CM and PM are in capillary contact with a water-saturated apoplast. A comparison of the mechanical properties of the CM and PM revealed that the hydrated PM was 3.3 times (apple) and 4.9 times (pear) more extensible than the CM, as indicated by the higher *ɛ*_max_ values (Table 2). This observation is consistent across experiments and across seasons (B. P. Khanal, unpubl. observ.). It could be argued that the higher extensibility of the PM is related to its higher *in vivo* strain and that the tensile test merely re-imposed the *in vivo* strain upon the relaxed specimen. However, this interpretation is considered unlikely. Instead, converting the biaxial strain released from the CM and PM discs to a uniaxial strain (assuming, as a first approximation, isotropic and ideal behaviour; Knoche *et al.* 2004) yields uniaxial strains that are close to the *ɛ*_max_ of the CM in apple (release of uniaxial strain 2.8 ± 0.2 % vs. *ɛ*_max_ of 2.9 ± 0.2 %) and pear (release of uniaxial strain 3.7 ± 0.3 % vs. *ɛ*_max_ of 3.4 ± 0.2 %; Tables 1 and 2). However, the calculated uniaxial strains were lower than the *ɛ*_max_ of the PM in the two species (release of uniaxial strain in apple 5.4 ± 0.3 % vs. *ɛ*_max_ of 9.6 ± 0.4 %; release of uniaxial strain in pear 10.4 ± 0.6 % vs. *ɛ*_max_ of 16.5 ± 0.5 %; Tables 1 and 2). This calculation shows that for the CM, the *in vivo* strain was close to or even exceeded the *ɛ*_max_ in the tensile test and, hence, the strain at failure. In contrast, the strains of the PM on the fruit skins *in vivo* were still below their respective *ɛ*_max_ values.

### Site of failure in a russetted fruit surface differs between apple and pear

The site of failure of a specimen composed of both the CM and PM differed between apple and pear. In apple the CM/PM borderline was the weakest point whereas in pear the CM itself failed more often. We do not fully understand the reason for this difference. Possible factors are: (i) the thinner CM in pear compared with apple, (ii) the more pronounced hypodermal development of the CM in pear compared with apple (B. P. Khanal, unpubl. observ.) and (iii) an uneven distribution of strain and, hence, the development of stress concentrations triggering an earlier CM failure.

### Cell layers underlying the CM and PM represent the principal load-bearing structures

The load-bearing structures of both apple and pear skins were the ES and the PS rather than the CM and PM. This conclusion is based on the observation that the *F*_max_ values of the ES and PS always exceeded those of the CM and PM. This is not surprising, since the primary load-bearing material of the ES and PS resides in the cellulosic cell walls of the underlying cell layers. Unfortunately, it was technically difficult to prepare ES or PS samples that were thinner than those investigated here, so we were unable to identify the relative contributions of the individual cell layer(s) to the mechanical properties of the whole ES and PS composites. However, the cells of the epidermal and hypodermal layers are the most likely candidates for a mechanical function because of their small size and thickened walls compared with the much larger, thin-walled cells of the parenchyma. The lower *ɛ*_max_ of the CM as compared with the ES is consistent with the observation that microscopic cracks in the CM are the first visible symptom of russeting (Faust and Shear 1972*a*).

## Conclusions and forward look

The most striking and consistent difference in the mechanical properties between the CM of a non-russeted surface and the PM of a russeted surface is the higher plasticity of the hydrated PM compared with the CM. This is a desirable property in the PM if it is to function as a ‘repair patch’ for an overly strained surface having a cracked CM. It allows the PM to cope with ongoing area expansions during growth without excessive stress concentration arising (Considine and Brown 1982). Thus, growth stresses are also distributed uniformly when the surface is a composite of the CM and PM. This is an important prerequisite for a regular-shaped fruit. Our results further demonstrate that *in vivo* the CM of both apple and pear (but not the PM) are strained to near their failure limits. If these limits are exceeded, failure in apple will occur at the CM/PM transition, resulting in a continuous spread of russeting on the expanding surface. In pear, however, the CM appears to be the weakest point, as failures occur primarily within the CM rather than in the PM or at the CM/PM interface.

## Sources of funding

This research was funded by a grant from the Niedersächsisches Ministerium für Wissenschaft und Kultur (grant no. 76251-17-4/09/ZN2543) within the WEGA Framework.

## Contributions by the authors

E.G. and M.K. obtained the funds to support the study. E.G., M.K. and B.P.K. planned the experiments. B.P.K. conducted the mechanical studies and E.G. and B.P.K. the microscopy work. B.P.K. and M.K. analysed the data and wrote the manuscript. B.P.K., E.G. and M.K. revised and edited the paper.

## Conflict of interest statement

None declared.
